# Avelumab monotherapy as first-line or second-line treatment in patients with metastatic renal cell carcinoma: phase Ib results from the JAVELIN Solid Tumor trial

**DOI:** 10.1186/s40425-019-0746-2

**Published:** 2019-10-24

**Authors:** Ulka Vaishampayan, Patrick Schöffski, Alain Ravaud, Christian Borel, Julio Peguero, Jorge Chaves, John C. Morris, Nuria Kotecki, Martin Smakal, Dongli Zhou, Silke Guenther, Marcis Bajars, James L. Gulley

**Affiliations:** 1 Karmanos Cancer Institute, Wayne State University, 4100 John R. Street, Detroit, MI 48201 USA; 2 General Medical Oncology, University Hospitals Leuven, Leuven Cancer Institute, Leuven, Belgium; 3 Medical Oncology, Bordeaux University Hospital, Bordeaux, France; 4 Medical Oncology, Centre Paul Strauss, Centre de Recherche, Centres de Lutte Contre le Cancer (CRLCC), Strasbourg, France; 5 Oncology Haematology, Oncology Consultants, Houston, USA; 6 Haematology-Oncology, Northwest Medical Specialties, Lakewood, USA; 7 Internal Medicine, University of Cincinnati, Cincinnati, USA; 8 Centre Oscar Lambret, Lille, France; 9 Horovice Oncology Clinic, Nemocnice Rudolfa a Stefanie Benešov, a. s, Benešov, Czech Republic; 10 Merck Serono Pharmaceutical R&D Co, Beijing, China; 11 Merck KGaA, Darmstadt, Germany; 12 Global Clinical Development, EMD Serono, Billerica, USA; 13 Genitourinary Malignancies Branch, Center for Cancer Research, National Cancer Institute, National Institutes of Health, Bethesda, USA

**Keywords:** Avelumab, PD-L1, Renal cell carcinoma, Metastatic, Phase I

## Abstract

**Background:**

Antibodies targeting programmed death-1 (PD-1) or programmed death-ligand 1 (PD-L1) have shown clinical activity in the treatment of metastatic renal cell carcinoma (mRCC). This phase Ib cohort of the JAVELIN Solid Tumor trial assessed the efficacy and safety of avelumab (anti–PD-L1) monotherapy in patients with mRCC as either first-line (1 L) or second-line (2 L) treatment.

**Methods:**

Patients with mRCC with a clear-cell component who were treatment naive (1 L subgroup) or had disease progression after one prior line of therapy (2 L subgroup) received avelumab 10 mg/kg intravenous infusion every 2 weeks. Endpoints included confirmed best overall response, duration of response (DOR), progression-free survival (PFS), overall survival (OS), PD-L1 expression, and safety.

**Results:**

A total of 62 patients were enrolled in the 1 L subgroup, and 20 patients were enrolled in the 2 L subgroup. In the 1 L and 2 L subgroups, confirmed objective response rates were 16.1 and 10.0%, median DOR was 9.9 months (95% confidence interval [CI], 2.8–not evaluable) and not evaluable (95% CI, 6.9–not evaluable), median PFS was 8.3 months (95% CI, 5.5–9.5) and 5.6 months (95% CI, 2.3–9.6), and median OS was not evaluable (95% CI, not evaluable) and 16.9 months (95% CI, 8.3–not evaluable), respectively. Treatment-related adverse events (TRAEs) of any grade occurred in 51 patients in the 1 L subgroup (82.3%) and 14 patients in the 2 L subgroup (70.0%). Grade ≥ 3 TRAEs occurred in eight patients in the 1 L subgroup (12.9%) and one patient in the 2 L subgroup (5.0%). No treatment-related deaths occurred.

**Conclusion:**

Avelumab showed clinical activity and a manageable safety profile in both the 1 L and 2 L treatment setting in patients with mRCC. These data support the use of avelumab in combination with other agents in mRCC.

**Trial registration:**

ClinicalTrials.gov: NCT01772004; registered 21 January, 2013.

## Background

Renal cell carcinoma (RCC) is the most common type of kidney cancer, with clear-cell RCC being the most common subtype [1]. Historically, metastatic RCC (mRCC) has had a poor prognosis, with an average 5-year survival rate of ≈11% [2]. Also, mRCC is highly resistant to chemotherapy and radiation treatment [3, 4]. In recent years, progress has been made in the treatment of advanced or metastatic RCC and multiple targeted therapies have been approved, including tyrosine kinase inhibitors (TKIs), mammalian target of rapamycin inhibitors), and the anti–vascular endothelial growth factor antibody bevacizumab in combination with interferon alpha [5]. These targeted therapies have shown clinical activity and prolonged survival in patients with mRCC [6]; however, responses are generally short-lived, development of treatment resistance is common [5, 7], and different classes of targeted therapy are associated with characteristic toxicity profiles that have implications for patient treatment selection [5].

In recent years, immune checkpoint inhibitors (ICIs) have become an established therapeutic class, with clinical activity seen in various tumor types [8, 9]. In RCC, the immune checkpoint protein programmed death-1 (PD-1) and its ligand (PD-L1) are widely expressed on immune cells that infiltrate the tumor microenvironment and tumor cells, respectively [10–12]. Moreover, increased PD-1/L1 expression in RCC is associated with aggressive pathological features and a worse prognosis [10–12]. In patients with mRCC, anti–PD-1 and anti–PD-L1 antibodies have shown promising responses and improved overall survival (OS), both as monotherapy and in combination with other classes of agents. Nivolumab (anti–PD-1) was the first agent in this class to be approved by regulatory authorities, based on findings from the randomized phase III CheckMate 025 trial, which compared nivolumab monotherapy with everolimus in patients with advanced RCC who had received prior antiangiogenic therapy [13]. More recently, nivolumab in combination with ipilimumab (anti–cytotoxic T-lymphocyte protein 4) was approved for patients with previously untreated, intermediate- or poor-risk, advanced RCC, based on OS data from the phase III CheckMate 214 trial of nivolumab plus ipilimumab compared with sunitinib [14].

Avelumab is a human IgG1 monoclonal antibody that binds PD-L1, inhibiting the interaction with PD-1 and restoring antitumor immune responses [15]. Avelumab has been approved in various countries for the treatment of metastatic Merkel cell carcinoma and advanced urothelial carcinoma that has progressed following platinum-containing therapy [16]. The large, phase I, multicohort JAVELIN Solid Tumor trial (> 1700 patients; NCT01772004) assessed avelumab monotherapy in various tumors [17–23]. Here we report the efficacy and safety data from the phase Ib cohort of patients with mRCC, including subgroups who received either first-line (1 L) or second-line (2 L) avelumab monotherapy. When this study was initiated, phase III data for an ICI (nivolumab) as a 2 L treatment for advanced RCC had been reported [13]; however, no data for 1 L ICI treatment had been reported, providing the rationale to investigate the clinical activity of avelumab in both the 1 L and 2 L treatment settings. Subsequently, studies of anti–PD-1/PD-L1 antibodies in combination with targeted therapies as 1 L treatment for advanced or metastatic RCC were reported [14, 24–27]; this includes trials of avelumab combined with axitinib, particularly the recently reported phase III JAVELIN Renal 101 study, which showed superior efficacy with this regimen compared with sunitinib, and led to the recent FDA approval of avelumab and axitinib in combination for the treatment of advanced RCC [16, 25, 26]. Pembrolizumab (anti–PD-1) in combination with axitinib has also been approved by the FDA [24]. By evaluating the activity of avelumab monotherapy, the current study provides context for the improved efficacy seen with avelumab plus axitinib.

## Methods

### Study design and patients

JAVELIN Solid Tumor is an international, multicohort, open-label, phase I trial. Key eligibility criteria for this phase Ib expansion cohort were adults with histologically or cytologically confirmed mRCC with a clear-cell component, an Eastern Cooperative Group performance status (ECOG PS) of 0 or 1 and measurable disease by Response Evaluation Criteria in Solid Tumors (RECIST) v1.1. Patients were enrolled irrespective of PD-L1 expression status and had received no prior treatment (1 L subgroup) or had disease progression after one prior line of metastatic therapy (2 L subgroup). Key exclusion criteria included prior treatment with a T-cell–targeting antibody/drug; other cancer diagnosis within 5 years prior to study entry; and known autoimmune disease or hypersensitivity to monoclonal antibodies. Full eligibility criteria have been reported [17].

The trial was conducted in accordance with the Declaration of Helsinki and the International Council for Harmonisation Guideline for Good Clinical Practice. The protocol was approved by the institutional review board or independent ethics committee of each centre; all patients provided written informed consent before enrolment.

### Treatment

All patients received avelumab 10 mg/kg by intravenous infusion every 2 weeks until disease progression, unacceptable toxicity, or other criteria for withdrawal were met (reported previously) [17]. Dose reductions were not permitted. Antihistamine premedication was given 30–60 min before each infusion. Grade 2 AEs were managed by treatment delays of up to two subsequent omitted doses; events that did not resolve to grade ≤ 1 or recurred resulted in permanent treatment discontinuation.

### Assessments

Clinical activity and safety were analyzed in all patients who received at least one dose of avelumab. Tumors were assessed every 6 weeks for the first year and every 12 weeks thereafter by investigators according to RECIST v1.1. Safety was assessed at each biweekly visit, and AEs were graded according to the National Cancer Institute’s Common Terminology Criteria for Adverse Events (NCI-CTCAE), v4.0. Immune-related AEs (irAEs) were identified using a prespecified list of Medical Dictionary for Regulatory Activities (MedDRA) preferred terms, followed by comprehensive medical review. Infusion-related reactions (IRRs) were identified using an expanded definition that included both a prespecified list of MedDRA preferred terms (IRR, drug hypersensitivity, or hypersensitivity reaction) that occurred after infusion on the same day or following day, and additional signs/symptoms that occurred on the day of infusion and resolved within 2 days. PD-L1 expression was assessed using a proprietary immunohistochemistry assay (PD-L1 IHC 73–10 assay; Dako, Carpinteria, CA). PD-L1+ status was defined as PD-L1 expression on ≥ 1% of tumor cells.

### Endpoints

Prespecified endpoints included confirmed best overall response according to RECIST v1.1 (investigator assessed), duration of response (DOR), progression-free survival (PFS) according to RECIST v1.1, OS, PD-L1 expression, and safety.

### Statistical analysis

Enrolment of the 1 L subgroup began after documentation of two objective responses in the 2 L subgroup. Separate analyses of 1 L and 2 L subgroups were prespecified. The planned sample size of 20 patients in the 2 L subgroup was selected to enable observation of at least two responders with a probability of > 89.8% if the true objective response rate (ORR; proportion of patients with a partial response [PR] or complete response [CR]) was ≥ 18%. The planned sample size of 60 patients in the 1 L subgroup was selected to provide 95% Clopper-Pearson confidence intervals (CIs) for an ORR of 20% (95% CI, 10.8–32.3) in the case of 12 responders and 25% (95% CI, 14.7–37.9) in the case of 15 responders. Time-to-event endpoints were estimated using the Kaplan-Meier method, and CIs for medians were calculated using the Brookmeyer-Crowley method. *P* values for the association between PD-L1 status and ORR were determined using Fisher exact test.

## Results

### Patients and treatment

Between May 11, 2015, and October 13, 2016, 82 patients were enrolled, comprising 62 in the 1 L subgroup and 20 in the 2 L subgroup (Table 1). In the 1 L and 2 L subgroups, respectively, median age was 62 years (range, 36–85) and 69 years (range, 30–80); 43 (69.4%) and 15 (75.0%) patients were male; 25 (40.3%) and 11 (55.0%) had an ECOG PS of 1; and 20 (32.3%) and four (20.0%) had PD-L1+ tumors. At the time of data cutoff (April 27, 2018), median follow-up in the 1 L and 2 L subgroups was 26.2 months (range, 18–29) and 34.1 months (range, 28–35), respectively. Median duration of treatment was 9.6 months (range, 0.9–29.0) in the 1 L subgroup and 5.3 months (range, 0.9–34.5) in the 2 L subgroup. At last follow-up, 12 patients (19.4%) in the 1 L subgroup and two patients (10.0%) in the 2 L subgroup remained on treatment. In both subgroups, the most common reason for discontinuation was disease progression (1 L, *n* = 40 [64.5%]; 2 L, *n* = 14 [70.0%]), and other reasons were AE (1 L, *n* = 4 [6.5%]; 2 L, *n* = 3 [15%]), withdrawal of consent (1 L, *n* = 1 [1.6%]), death (1 L, *n* = 1 [1.6%]; 2 L, *n* = 1 [5.0%]), and other (1 L, *n* = 4 [6.5%]; two patients required prohibited concomitant medication, one patient met an exclusion criterion, and one patient decided to undergo surgery).

**Table 1:** Patient baseline characteristics

Characteristics	1 L (*n* = 62)	2 L (*n* = 20)
Age, *n* (%)		
< 65 years	37 (59.7)	7 (35.0)
≥ 65 years	25 (40.3)	13 (65.0)
Median age (range), years	62 (36–85)	69 (30–80)
Sex, *n* (%)		
Male	43 (69.4)	15 (75.0)
Female	19 (30.6)	5 (25.0)
ECOG PS, *n* (%)		
0	37 (59.7)	9 (45.0)
1	25 (40.3)	11 (55.0)
MSKCC prognostic risk group, *n* (%)		
Favorable	2 (3.2)	0
Intermediate	53 (85.5)	17 (85.0)
Poor	7 (11.3)	3 (15.0)
IMDC prognostic risk group, *n* (%)		
Favorable	24 (38.7)	5 (25.0)
Intermediate	27 (43.5)	13 (65.0)
Poor	11 (17.7)	2 (10.0)
Median time since diagnosis of metastatic disease (range), months	2.5 (0.4–90.4)	15.0 (1.6–80.4)
Number of prior anticancer therapy lines for metastatic or locally advanced disease, *n* (%)		
0	62 (100.0)^a^	0
1	0	19 (95.0)
2	0	0
3	0	0
≥ 4	0	1 (5.0)
PD-L1 status (≥ 1% tumor cells), *n* (%)		
Positive	20 (32.3)	4 (20.0)
Negative	21 (33.9)	9 (45.0)
Not evaluable	21 (33.9)	7 (35.0)

^a^ One patient (1.6%) received prior adjuvant therapy

*1 L* first-line subgroup, *2 L* second-line subgroup, *ECOG PS* Eastern Cooperative Oncology Group performance status, *MSKCC* Memorial Sloan-Kettering Cancer Center, *IMDC* International Metastatic Renal Cell Carcinoma Database Consortium, *PD-L1* programmed death-ligand 1

### Antitumor activity

**Fig. 1:**
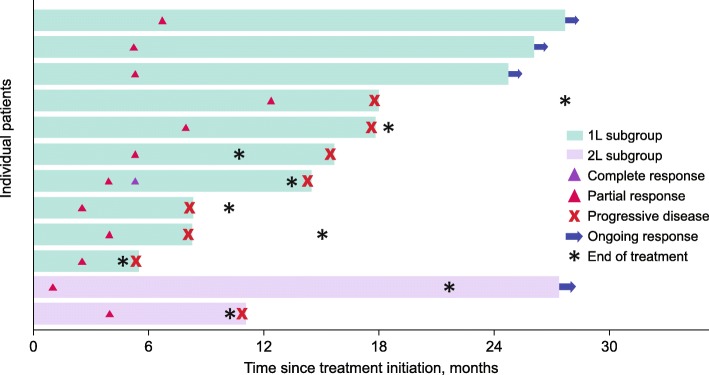
Time to and duration of confirmed response. *1 L* first-line, *2 L* second-line

**Fig. 2:**
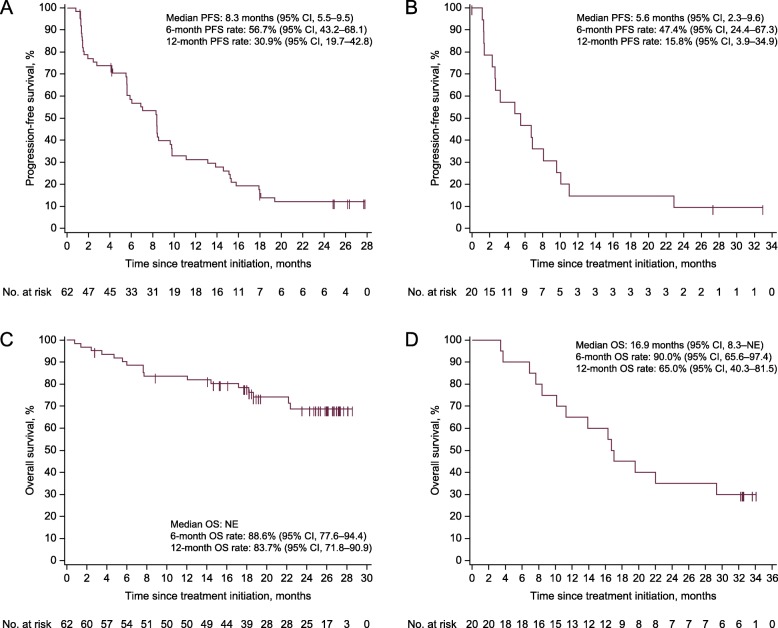
Kaplan-Meier estimates of progression-free survival (PFS) and overall survival (OS). **a** PFS in the first-line (1 L) subgroup. **b** PFS in the second-line (2 L) subgroup. **c** OS in the 1 L subgroup. **d** OS in the 2 L subgroup. *CI* confidence interval, *NE* not evaluable

In the 1 L and 2 L subgroups, respectively, the ORR was 16.1% (CR, *n* = 1 [1.6%]; PR, *n* = 9 [14.5%]) and 10.0% (PR, *n* = 2) (Table 2**;** Fig. 1); median DOR was 9.9 months (95% CI, 2.8–not evaluable) and not evaluable (95% CI, 6.9–not evaluable); and 38 (61.3%) and 13 (65.0%) patients had a best overall response of stable disease, resulting in disease control rates of 77.4 and 75.0%. Median PFS was 8.3 months (95% CI, 5.5–9.5) in the 1 L subgroup and 5.6 months (95% CI, 2.3–9.6) in the 2 L subgroup (Fig. 2); 6-month and 12-month PFS rates were 56.7 and 30.9% in the 1 L subgroup, and 47.4 and 15.8% in the 2 L subgroup, respectively. In the 1 L and 2 L subgroups, median OS was not evaluable (95% CI, not evaluable) and 16.9 months (95% CI, 8.3–not evaluable); 6-month and 12-month OS rates were 88.6 and 83.7%, and 90.0 and 65.0%, in the 1 L and 2 L subgroups, respectively.

**Table 2:** Confirmed objective responses

Response	1 L (*n* = 62)	2 L (*n* = 20)
Best overall response, *n* (%)		
Complete response	1 (1.6)	0
Partial response	9 (14.5)	2 (10.0)
Stable disease	38 (61.3)	13 (65.0)
Progressive disease	11 (17.7)	4 (20.0)
Not evaluable	3 (4.8)^a^	1 (5.0)^b^
Objective response rate (95% CI), %	16.1 (8.0–27.7)	10.0 (1.2–31.7)
Disease control rate, %	77.4	75.0
Response duration	1 L (*n* = 10)	2 L (*n* = 2)
Median duration of response (95% CI), months	9.9 (2.8–NE)	NE (6.9–NE)
Proportion of patients with specified duration of response (95% CI), %^c^		
6 months	60.0 (25.3–82.7)	100.0 (NE)
12 months	30.0 (7.1–57.8)	50.0 (0.6–91.0)

^a^ Due to no postbaseline assessment (*n* = 2) or stable disease of insufficient duration (< 6 weeks after start date without further tumor assessment; *n* = 1)

^b^ All postbaseline assessments not evaluable (*n* = 1)

^c^ Based on Kaplan-Meier estimates

*1 L* first-line subgroup, *2 L* second-line subgroup, *CI* confidence interval, *NE* not evaluable

### Biomarker subgroup analysis

Among evaluable patients in the 1 L subgroup with PD-L1+ (*n* = 20) or PD-L1− (*n* = 21) tumors, respectively, the ORR was 10.0% (95% CI, 2.7–24.5) and 14.3% (95% CI, 5.4–29.1), median PFS was 5.8 months (95% CI, 1.9–13.0) and 8.3 months (95% CI, 5.5–15.1), 6-month PFS rates were 48.5% (95% CI, 25.4–68.2) and 66.7% (95% CI, 42.5–82.5), median OS was not evaluable in either group*,* and 12-month OS rates were 85.0% (95% CI, 60.4–94.9) and 90.5% (95% CI, 67.0–97.5) (Additional file 1). Results from the 2 L subgroup are not reported owing to low patient numbers.

### Safety

Of patients in the 1 L and 2 L subgroups, 51 (82.3%) and 14 (70.0%) had a treatment-related AE (TRAE) of any grade, including eight (12.9%) and one (5.0%) who had a grade ≥ 3 TRAE, respectively (Table 3; Additional file 2). The only grade ≥ 3 TRAE that occurred in more than one patient was increased lipase (1 L, *n* = 4 [6.5%]). TRAEs led to discontinuation in three patients (4.8%) in the 1 L subgroup (anaphylactic reaction, aspartate aminotransferase increase, and nephritis) and two patients (10.0%) in the 2 L subgroup (IRR and pneumonitis). IRRs (based on an expanded definition) occurred in 22 patients (35.5%) in the 1 L subgroup and six patients (30.0%) in the 2 L subgroup; all were grade 1 or 2. Of patients in the 1 L and 2 L subgroups, 18 (29.0%) and three (15.0%) had an irAE of any grade, respectively. The most commonly occurring irAEs (≥ 10% in either subgroup) were thyroid disorders (1 L, *n* = 10 [16.1%]; 2 L, *n* = 2 [10.0%] and immune-related rash (1 L, *n* = 9 [14.5%]; 2 L, *n* = 1 [5.0%]). Two patients (3.2%) in the 1 L subgroup had a grade 3 irAE (rash and colitis, both *n* = 1); no patients in the 2 L subgroup had a grade 3 irAE. No grade 4 irAEs occurred in either subgroup. In the 1 L and 2 L subgroups, respectively, fourteen patients (22.6%) and seven patients (35.0%) had serious AEs, which were related to treatment in two patients (3.2%) in the 1 L subgroup (grade 3 colitis and grade 2 hyperthermia, both *n* = 1). Four patients (6.5%) in the 1 L subgroup and two patients (10.0%) in the 2 L subgroup had an AE leading to death (none treatment related).

**Table 3:** Incidence of treatment-related adverse events (TRAEs), infusion-related reactions (IRRs), and immune-related adverse events (irAEs)

	1 L (*n* = 62)		2 L (*n* = 20)	
	Any Grade	Grade ≥ 3	Any Grade	Grade ≥ 3
Any TRAE, *n* (%)^a, b^	51 (82.3)	8 (12.9)	14 (70.0)	1 (5.0)
Pruritus	12 (19.4)	0	0	0
Fatigue	11 (17.7)	0	5 (25.0)	1 (5.0)
Asthenia	9 (14.5)	0	1 (5.0)	0
Nausea	9 (14.5)	0	0	0
Diarrhea	8 (12.9)	0	3 (15.0)	0
Pyrexia	8 (12.9)	0	2 (10.0)	0
Decreased appetite	6 (9.7)	0	2 (10.0)	0
Increased lipase	6 (9.7)	4 (6.5)	1 (5.0)	0
Rash	6 (9.7)	1 (1.6)	0	0
Pneumonitis	2 (3.2)	0	2 (10.0)	0
Anaphylactic reaction	1 (1.6)	1 (1.6)	0	0
Colitis	1 (1.6)	1 (1.6)	0	0
Thrombocytopenia	1 (1.6)	1 (1.6)	0	0
Infusion-related reactions, *n* (%)^c, d^	22 (35.5)	0	6 (30.0)	0
Any immune-related AE, *n* (%)^c^	18 (29.0)	2 (3.2)	3 (15.0)	0
Hypothyroidism	7 (11.3)	0	1 (5.0)	0
Rash	5 (8.1)	1 (1.6)	0	0
Hyperthyroidism	3 (4.8)	0	0	0
Pruritus	3 (4.8)	0	0	0
Blood TSH increased	2 (3.2)	0	1 (5.0)	0
Colitis	1 (1.6)	1 (1.6)	0	0
Diarrhea	1 (1.6)	0	0	0
Erythema	1 (1.6)	0	0	0
Nephritis	1 (1.6)	0	0	0
Pruritus generalized	1 (1.6)	0	0	0
Psoriasis	1 (1.6)	0	0	0
Rash generalized	1 (1.6)	0	0	0
Rash macular	1 (1.6)	0	0	0
Pneumonitis	0	0	1 (5.0)	0
Rash pruritic	0	0	1 (5.0)	0

^a^ The incidence of treatment-related infusion-related reactions based on the single MedDRA preferred term is not listed

^b^ Any grade TRAEs in ≥ 10% patents and all grade 3 TRAEs

^c^ Composite term; includes AEs categorized as infusion-related reaction, drug hypersensitivity, or hypersensitivity reaction that occurred on the day of infusion or day after infusion, in addition to signs/symptoms of infusion-related reaction (based on a prespecified list of MedDRA preferred terms) that occurred on the same day of infusion and resolved within 2 days

^d^ Includes AEs classified by investigators as related or unrelated to treatment

*1 L* first-line subgroup, *2 L* second-line subgroup, *AE* adverse event, *MedDRA* Medical Dictionary for Regulatory Activities, *TRAE* treatment-related adverse events, *TSH* thyroid-stimulating hormone

## Discussion

In this phase Ib study, avelumab monotherapy showed clinical activity as a 1 L or 2 L treatment for patients with mRCC. Responses were durable (median DOR was 9.9 months [1 L] and not evaluable [2 L]), and disease control rates were high in both subgroups (1 L, 77.4%; 2 L, 75.0%). Median PFS was 8.3 months in the 1 L subgroup and 5.6 months in the 2 L subgroup, and 12-month OS rates were 83.7% (1 L; median, not evaluable) and 65.0% (2 L; median, 16.9 months). Responses to avelumab occurred irrespective of PD-L1 status, and no significant survival difference was seen between PD-L1+ and PD-L1− populations. Avelumab showed an acceptable safety profile, including a low rate of grade 3/4 TRAEs (12.9 and 5.0% in the 1 L and 2 L subgroups, respectively). These results are comparable with those reported with TKI monotherapy [7].

Results of this study were generally consistent with those in previous studies of anti–PD-1/PD-L1 monotherapy administered as either 1 L or 2 L treatment for mRCC. In the nivolumab monotherapy arm of the phase III CheckMate 025 study (patients with previously treated advanced clear-cell RCC [*n* = 410]), median PFS was 4.6 months (95% CI, 3.7–5.4), median OS was 25 months (95% CI, 21.8–not evaluable), the ORR was 25%, and 19% of patients had a grade 3/4 TRAE [13]. In the atezolizumab monotherapy arm of the randomized, phase II IMmotion150 study of patients with treatment-naive mRCC (*n* = 103), median PFS was 6.1 months (95% CI, 5.4–13.6), OS was not reported, the ORR was 25% (CR, 11%; PR, 14%), and 17% of patients had a grade 3/4 TRAE [28]. Finally, in cohort A of the phase II KEYNOTE-427 study, which enrolled patients with advanced clear-cell RCC (*n* = 110), 1 L pembrolizumab monotherapy resulted in a median PFS of 6.9 months (95% CI, 5.1–not evaluable), 6-month OS rate of 92.4% (median, not reached), and ORR of 33.6% (95% CI, 24.8–43.4), and 18.2% of patients had a grade 3–5 TRAE [29].

Preliminary findings from this study supported the rationale for the JAVELIN Renal 100 study (phase Ib avelumab in combination with axitinib [*n* = 55]) [25], and the recently reported JAVELIN Renal 101 trial, a randomized phase III study of avelumab plus axitinib (*n* = 442) compared with sunitinib (*n* = 444) as 1 L treatment for patients with advanced clear-cell RCC. Median PFS in patients with PD-L1+ tumors (primary endpoint) was 13.8 vs 7.2 months, respectively (hazard ratio, 0.61 [95% CI, 0.47–0.79]; *P* < 0.001); in all patients (irrespective of PD-L1 expression), median PFS was 13.8 vs 8.4 months (hazard ratio, 0.69 [95% CI, 0.56–0.84]; *P* < 0.001), and the ORR was 51.4% vs 25.7%, respectively [26]. The enhanced efficacy of the combination may result from synergistic antitumor effects provided by the different mechanisms of action of avelumab and axitinib, including the known immunomodulatory effects of vascular endothelial growth factor receptor tyrosine kinase inhibitors [25, 26]. Improved efficacy with anti–PD-1/PD-L1 combinations in the 1 L setting have also been reported for pembrolizumab plus axitinib (KEYNOTE-426) [24], nivolumab plus ipilimumab (CheckMate 214) [14], and atezolizumab plus bevacizumab (IMmotion151) [30], highlighting the rapidly evolving treatment landscape in advanced RCC.

## Conclusion

In conclusion, results from this study show the efficacy and safety of avelumab in patients with mRCC, supporting the foundational role of ICIs within combination treatment regimens for this disease.

## Supplementary information

**Additional file 1 **Kaplan-Meier estimates of **a** progression-free survival (PFS) and **b** overall survival (OS) in the first-line subgroup according to programmed death-ligand 1 (PD-L1) status (based on expression in ≥1% of tumor cells). *CI* confidence interval, *NE* not evaluable. Kaplan-Meier estimates of progression-free survival and overall survival for patients in the first-line subgroup according to programmed death-ligand 1 status.

**Additional file 2.** Overview of key safety outcomes. Key safety outcomes for patients in both the first-line and second-line treatment setting.

## Data Availability

For all new products or new indications approved in both the European Union and the United States after January 1, 2014, Merck KGaA, Darmstadt, Germany will share patient-level and study-level data after deidentification, as well as redacted study protocols and clinical study reports from clinical trials in patients. These data will be shared with qualified scientific and medical researchers, upon researcher’s request, as necessary for conducting legitimate research. Such requests must be submitted in writing to the company’s data sharing portal. More information can be found at https://www.merckgroup.com/en/research/our-approach-to-research-and-development/healthcare/clinical-trials/commitment-responsible-data-sharing.html. Where Merck KGaA has a co-research, co-development or co-marketing/co-promotion agreement or where the product has been out-licensed, it is recognized that the responsibility for disclosure may be dependent on the agreement between parties. Under these circumstances, Merck KGaA will endeavor to gain agreement to share data in response to requests.

## Abbreviations

1 L: First-line
2 L: Second-line
AE: Adverse event
CI: Confidence interval
CR: Complete response
DOR: Duration of response
ECOG PS: Eastern Cooperative Oncology Group performance score
ICI: Immune-checkpoint inhibitor
irAE: Immune-related adverse event
IRR: Infusion-related reaction
MedDRA: Medical Dictionary for Regulatory Activities
mRCC: Metastatic renal cell carcinoma
NCI-CTCAE: National Cancer Institute Common Terminology Criteria for Adverse Events
ORR: Objective response rate
OS: Overall survival
PD-1: Programmed death-1
PD-L1: Programmed death-ligand 1
PFS: Progression-free survival
PR: Partial response
RCC: Renal cell carcinoma
RECIST: Response Evaluation Criteria In Solid Tumors
TKI: Tyrosine kinase inhibitor
TRAE: Treatment-related adverse event
