# Supplementary material for: Avelumab monotherapy as first-line or second-line treatment in patients with metastatic renal cell carcinoma: phase Ib results from the JAVELIN Solid Tumor trial
Source: J Immunother Cancer. 2019 Oct 24;7:275. doi: 10.1186/s40425-019-0746-2 (PMC6813090; doi:10.1186/s40425-019-0746-2)

**ADDITIONAL FILES**

**Additional File 1.** Kaplan-Meier estimates of **a** progression-free survival (PFS) and **b** overall survival (OS) in the first-line subgroup according to programmed death-ligand 1 (PD-L1) status (based on expression in ≥ 1% of tumor cells). *CI* confidence interval, *NE* not evaluable
**A**


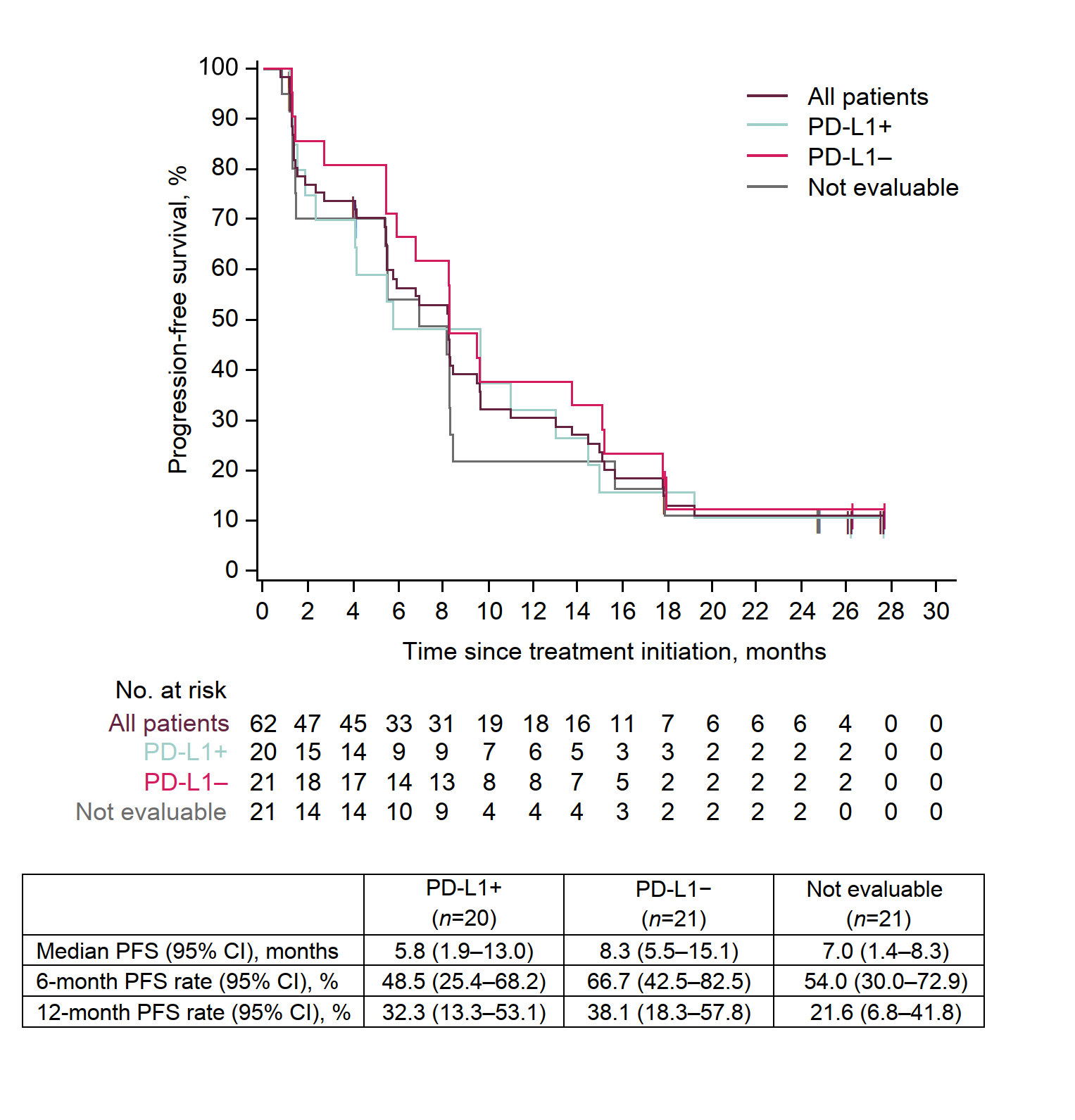


**B**


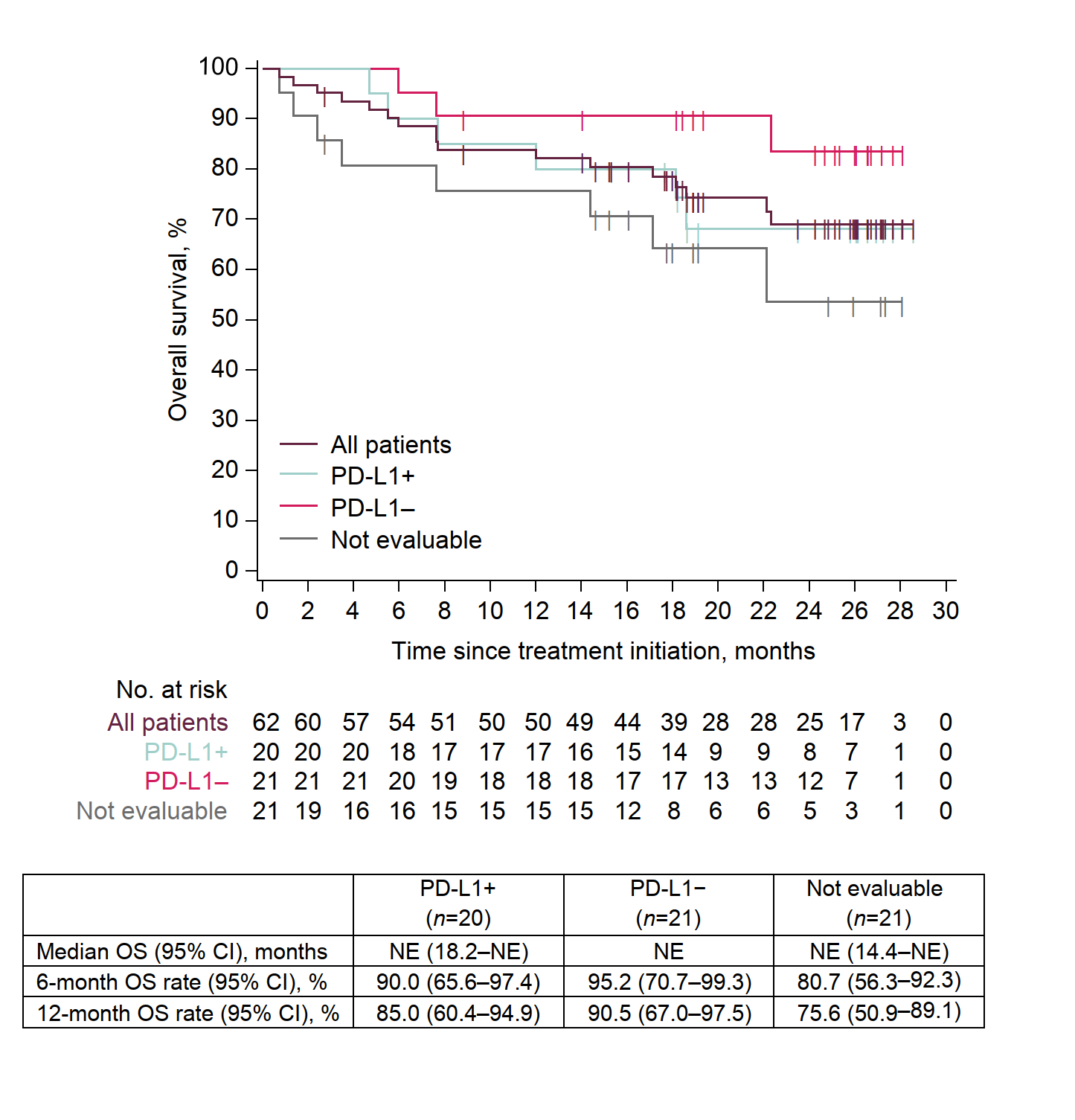

Supplement: Supplementary file 1 — Additional file 1 Kaplan-Meier estimates of a progression-free survival (PFS) and b overall survival (OS) in the first-line subgroup according to programmed death-ligand 1 (PD-L1) status (based on expression in ≥1% of tumor cells). CI confidence interval, NE not evaluable. Kaplan-Meier estimates of progression-free survival and overall survival for patients in the first-line subgroup according to programmed death-ligand 1 status. [file 40425_2019_746_MOESM1_ESM.docx]
