# Supplementary material for: Avelumab monotherapy as first-line or second-line treatment in patients with metastatic renal cell carcinoma: phase Ib results from the JAVELIN Solid Tumor trial
Source: J Immunother Cancer. 2019 Oct 24;7:275. doi: 10.1186/s40425-019-0746-2 (PMC6813090; doi:10.1186/s40425-019-0746-2)
Supplement: Supplementary file 2 — Additional file 2. Overview of key safety outcomes. Key safety outcomes for patients in both the first-line and second-line treatment setting. [file 40425_2019_746_MOESM2_ESM.docx]

**Additional File 2.** Overview of key safety outcomes

| **Patients, *n* (%)** | **1 L (*n* = 62)** | **2 L (*n* = 20)** |
| --- | --- | --- |
| AEs, any grade | 62 (100.0) | 19 (95.0) |
| AEs, grade ≥ 3 | 30 (48.4) | 13 (65.0) |
| TRAEs, any grade | 51 (82.3) | 14 (70.0) |
| TRAEs, grade ≥ 3 | 8 (12.9) | 1 (5.0) |
| AEs leading to treatment discontinuation | 3 (4.8) | 4 (20.0) |
| TRAEs leading to treatment discontinuation | 3 (4.8) | 2 (10.0) |
| Infusion-related reactions, any grade^a, b^ | 22 (35.5) | 6 (30.0) |
| Infusion-related reactions, grade ≥ 3^a, b^ | 0 | 0 |
| Immune-related AEs, any grade^b^ | 18 (29.0) | 3 (15.0) |
| Immune-related AEs, grade ≥ 3^b^ | 2 (3.2) | 0 |
| Serious AEs | 14 (22.6) | 7 (35.0) |
| Serious TRAEs | 2 (3.2) | 0 |
| AEs leading to death | 4 (6.5) | 2 (10.0) |
| TRAEs leading to death | 0 | 0 |

^a^ Composite term; includes AEs categorized as infusion-related reaction, drug hypersensitivity, or hypersensitivity reaction that occurred on the day of infusion or day after infusion, in addition to signs/symptoms of infusion-related reaction (based on a prespecified list of MedDRA preferred terms) that occurred on the same day of infusion and resolved within 2 days
^b^ Includes AEs classified by investigators as related or unrelated to treatment

*1L* first-line subgroup, *2L* second-line subgroup, *AE* adverse event, *MedDRA* Medical Dictionary for Regulatory Activities, *TRAE* treatment-related adverse event
